# Severe hypocapnia-induced coma in an asthma patient: When oxygen therapy becomes a double-edged sword

**DOI:** 10.5339/qmj.2026.17

**Published:** 2026-03-22

**Authors:** Waheeb Naser, Yavuz Yigit

**Affiliations:** 1 Hamad Medical Corporation, Doha, Qatar *Email: wnaser@hamad.qa

**Keywords:** Asthma, functional hyperventilation, coma, hypocapnia, respiratory alkalosis, oxygen therapy

## Abstract

**Background:**

Acute asthma exacerbations commonly lead to hyperventilation-induced hypocapnia (low arterial CO_2_ levels). While mild hypocapnia is frequent, extreme hypocapnia (PaCO_2_ ≤10 mmHg) causing coma is rare. This case highlights a life-threatening instance of severe hypocapnia in an asthma patient, complicated by anxiety-related hyperventilation and management challenges related to oxygen therapy.

**Case presentation:**

A 43-year-old man with a history of bronchial asthma presented to the emergency department with flu-like symptoms and acute shortness of breath. On arrival, he was normoxic, but he subsequently developed marked hyperventilation accompanied by carpopedal spasm and a state of unresponsiveness. Arterial blood gas analysis, while on supplemental oxygen, revealed profound respiratory alkalosis, with a pH of 7.75, PaCO_2_ of 10 mmHg, and PaO_2_ of 237 mmHg. Management consisted of cautious oxygen titration, controlled rebreathing to gradually restore carbon dioxide levels, light sedation to alleviate hyperventilation, and correction of electrolyte imbalances. Neuroimaging excluded intracranial pathology. The patient’s neurological status improved within hours, leading to complete recovery without the need for invasive mechanical ventilation.

**Discussion:**

Asthma exacerbations complicated by severe hypocapnia present a unique and often underrecognized therapeutic dilemma. Although oxygen is essential to prevent hypoxemia, excessive administration may further lower PaCO_2_, exacerbate cerebral vasoconstriction, and impair perfusion. Anxiety-related hyperventilation can further amplify these effects, potentially leading to transient cerebral dysfunction. Prompt recognition of extreme hypocapnia, careful modulation of oxygen delivery, and physiologically guided ventilation are essential to restore cerebral and systemic homeostasis.

**Conclusion:**

This case emphasizes the need to recognize extreme hypocapnia as a potential cause of coma in asthma exacerbations, especially when anxiety-driven hyperventilation is involved. With timely supportive care and careful ventilatory management, balancing oxygen therapy and CO_2_ control, full neurological recovery and clinical stability were achieved.

## 1. INTRODUCTION

Acute asthma exacerbations frequently lead to hyperventilation and consequent hypocapnia due to compensatory respiratory effort. Typically, patients with acute asthma present with Partial Pressure of Carbon Dioxide (PaCO_2_) levels ranging between 25 and 30 mmHg, and fewer than 10% demonstrate hypercapnia (PaCO_2_ >45 mmHg) upon initial assessment.^1^ Hypocapnia is a hallmark feature of acute asthma exacerbation, representing hyperventilation secondary to increased airway resistance and respiratory distress.^2^

In most severe asthma exacerbations, however, this early hypocapnia may not persist. As the disease progresses, respiratory muscles may fatigue due to the sustained increased work of breathing, resulting in reduced alveolar ventilation and a subsequent rise in PaCO_2_ toward normocapnia or hypercapnia. This transition is a critical warning sign of clinical deterioration and often signals impending ventilatory failure that may necessitate mechanical ventilation.^3^ Nonetheless, hypercapnia alone does not universally require intubation, as some patients can still stabilize with aggressive medical management, including bronchodilators, corticosteroids, and noninvasive support.^4,5^

While mild hypocapnia is generally well tolerated, extreme hypocapnia (PaCO_2_ <10 mmHg) is rare and may have serious systemic and neurological consequences. Severe respiratory alkalosis induces cerebral vasoconstriction, leading to a significant reduction in cerebral blood flow and resulting in symptoms such as dizziness, syncope, seizures, and coma.^6^ Additionally, alkalemia shifts the oxyhemoglobin dissociation curve to the left, impairing tissue oxygen delivery despite adequate arterial oxygenation. Severe hypocapnia may also trigger hypocalcemia, neuromuscular irritability, and cardiac complications, including arrhythmias and ischemia, due to heightened adrenergic sensitivity and myocardial vasoconstriction.^7^

This article presents a rare clinical case of asthma exacerbation accompanied by anxiety-induced hyperventilation, resulting in profound hypocapnia and transient coma. The report highlights the significant diagnostic and therapeutic challenges associated with extreme respiratory alkalosis. Written informed consent for publication was obtained from the patient, and institutional approval was granted by the Medical Research Center (MRC) under protocol number MRC-4-25-510.

## 2. CASE PRESENTATION

A 43-year-old male with a known history of asthma since adulthood, not on regular inhaler therapy and not under regular follow-up. presented to the Emergency Department at Hazm Mebaireek General Hospital (HMGH), a secondary care facility serving adult male patients in Doha’s Industrial Area. HMGH is part of Hamad Medical Corporation (HMC), Qatar’s main public healthcare provider, delivering specialized and emergency medical services within an integrated hospital network.^8^ He reported a three-day history of mild fever, cough, runny nose, and progressively worsening shortness of breath. He denied other comorbidities or prior intensive care admissions.

On arrival, his vital signs were: temperature 36.8°C, heart rate (HR) 116 beats per minute (bpm), respiratory rate (RR) 24 breaths/min, blood pressure (BP) 131/84 mmHg, and oxygen saturation (SpO_2_) 100% on room air. On examination by the attending physician 30 min later, the patient had almost the same vital signs, was conscious, alert, and oriented. He appeared mildly anxious but was able to speak in full sentences. Chest auscultation revealed mild bilateral wheezing. No use of accessory muscles, stridor, or altered mental status was noted at presentation. His temperature was 36.87°C, HR 89 bpm, RR 25 breaths/min, BP 133/95 mmHg, and SpO_2_ 100% on room air. Treatment was initiated with nebulized bronchodilators (salbutamol, budesonide, and ipratropium) along with intravenous hydrocortisone (200 mg). Initial investigations included venous blood gas (VBG), complete blood count (CBC), basic metabolic panel, viral panel, chest X-ray, and electrocardiogram (ECG). VBG showed: pH 7.51, PaCO_2_ 36 mmHg, PaO_2_ 27 mmHg, HCO_3_⁻ 28.7 mEq/L, and lactate 2.4 mmol/L.

Within an hour of presentation, his condition suddenly deteriorated, manifesting severe hyperventilation, carpopedal spasm, cold extremities, staring episodes, tearing, and eventual unresponsiveness, even to painful stimuli. Vital signs worsened: HR increased to 142 bpm, RR rose to 40 breaths per minute, BP elevated to 181/102 mmHg, and SpO_2_ dropped to 80% on room air.

### 2.1. Management dilemma

The critical challenge in management was determining the optimal oxygen therapy strategy. Excess oxygen administration risked worsening carbon dioxide washout and inducing cerebral vasoconstriction, while insufficient oxygenation posed a risk of hypoxemia. High-flow oxygen via a non-rebreather mask was initially administered for desaturation. but arterial blood gas (ABG) revealed severe respiratory alkalosis: pH 7.75, PaCO_2_ 10 mmHg, PaO_2_ 237 mmHg, HCO_3_− 13.9 mEq/L, lactate 6.30 mmol/L, and ionized calcium 1.07 mmol/L (Table 1). Considering these findings, supplemental oxygen was discontinued, and controlled rebreathing via a surgical mask (without oxygen flow) was implemented. The patient was managed conservatively with controlled ventilation, along with intravenous diphenhydramine (50 mg) and calcium gluconate (10 mL) for tetany and restlessness. Intubation equipment was prepared at the bedside; however, invasive intervention was not required.

### 2.2. Outcome and recovery

Fifteen minutes after initiation of the breathing technique without supplemental oxygen and conservative management, the patient showed progressive stabilization. His extremities relaxed, RR slowed, and vital signs began to normalize. A repeat VBG at 60 min confirmed correction of the metabolic derangements: pH 7.40, PaCO_2_ 38 mmHg, PaO_2_ 93 mmHg, HCO_3_⁻ 28 mEq/L, lactate 2.3 mmol/L, and ionized calcium 1.4 mmol/L. Neurologically, he gradually improved from unresponsiveness to a Glasgow Coma Scale (GCS) score of 8/15, with sluggish pupillary reactions.

Neuroimaging with computed tomography (CT) of the brain excluded acute pathology. Laboratory investigations, chest radiography, and ECG were unremarkable. The patient was admitted to the intensive care unit, where he regained full consciousness within 6–8 h. A respiratory viral panel was positive for rhinovirus. On the following day, he was stepped down to the general medical ward. Magnetic resonance imaging (MRI) of the brain revealed no abnormalities, and neurology reassessment recommended outpatient follow-up with an electroencephalogram (EEG). He was discharged on day four with a comprehensive asthma control plan.

## 3. DISCUSSION

Arterial PaCO_2_ trends in acute asthma exacerbations are essential for clinical assessment. Initial hypocapnia reflects compensatory hyperventilation; however, as respiratory muscle fatigue progresses, PaCO_2_ may drift toward normocapnia or hypercapnia, signaling impending ventilatory failure and the need for prompt intervention. Importantly, hypercapnia alone does not mandate intubation, as some patients may still respond to aggressive medical therapy.^9,10^

Severe alkalemia with extreme hypocapnia (PaCO_2_ <10 mmHg), although rare, is associated with severe multi-system complications. It reduces cerebral perfusion, shifts the oxyhemoglobin dissociation curve leftward, and accelerates anaerobic glycolysis. Furthermore, adrenergic hypersensitivity increases the risk of tachyarrhythmias and myocardial ischemia. Sepúlveda et al. described a 37-year-old woman who developed profound respiratory alkalosis (pH 7.68, PaCO₂ 10 mmHg) secondary to psychogenic hyperventilation during SARS-CoV-2 infection. Her condition progressed to coma, accompanied by lactic acidosis, ischemic ECG changes, tetany, and impaired tissue perfusion despite normal oxygenation.^11^ This case illustrates that functional hyperventilation syndromes may evolve into life-threatening organic consequences if not promptly identified. Similarly, our patient experienced profound alkalemia, resulting in altered consciousness, neuromuscular irritability, and lactic acidosis.

The spectrum of acid–base changes in asthma has been well described: Vasileiadis et al. reported that mild hypocapnia with respiratory alkalosis is a frequent early finding in acute asthma, whereas progression toward hypercapnia is more typical in severe or refractory cases.^12^ Raimondi et al. further analyzed acid–base disturbances in acute severe asthma and demonstrated that profound alkalemia of the degree observed in our patient is exceptionally uncommon.^2^ Gur et al. showed that extreme alkalemia on presentation to the emergency department is independently associated with poor outcomes, underscoring the prognostic significance of such derangements.^13^ Taken together, these reports support the rarity of our patient’s presentation and highlight the importance of rapid recognition and intervention in cases of extreme alkalemia.

Management in such cases remains challenging, particularly regarding oxygen supplementation. Excessive oxygen may blunt respiratory drive and exacerbate hypocapnia, whereas insufficient oxygen risks tissue hypoxia. In both our patient and the case reported by Sepúlveda et al.^11^ cautious oxygen titration, combined with sedation and supportive therapy, achieved full recovery without the need for invasive ventilation. This case emphasizes the importance of recognizing anxiety-driven hyperventilation as a potential aggravating factor in asthma-related respiratory alkalosis. Early recognition is crucial, as optimal management requires a nuanced approach balancing adequate oxygen delivery while avoiding further hypocapnia and its systemic consequences.

## 4. CONCLUSION

In this case, prompt recognition of extreme hypocapnia, cautious titration of oxygen therapy, and individualized supportive care—including controlled ventilation, light sedation, and correction of electrolyte imbalances—were essential for the patient’s favorable neurological recovery and positive clinical outcome. Extreme hypocapnia should be recognized as a reversible cause of coma in asthma exacerbations. Early identification and tailored physiological management are crucial for achieving optimal neurological and clinical results.

## COMPETING INTERESTS

The authors have no conflicts of interest to declare.

## Figures and Tables

**Table 1:** Patient’s arterial blood gases at deterioration.

Parameter	Value	Unit	Reference/notes
pH	7.75 ↑	–	Severe alkalemia
pCO_2_	10 ↓	mmHg	Respiratory alkalosis
pO_2_	237 ↑	mmHg	On oxygen
Na^+^	135	mmol/L	Borderline low
K^+^	3.6	mmol/L	Normal
Cl^−^	107	mmol/L	Normal
Ca^²+^ (ionized)	1.07 ↓	mmol/L	Low
Glucose	6.0	mmol/L	Mildly elevated
Lactate	6.3 ↑	mmol/L	High (lactic acidosis despite alkalemia)
**Co-Oximetry**			
O_2_Hb	97.6	%	Good saturation
COHb	1.2	%	Normal
MetHb	1.1	%	Normal
HHb	0.1	%	Very low
SpO_2_	99.9	%	Oxygen saturation
**Derived Parameters**			
TCO_2_	14.2 ↓	mmol/L	Low
BEecf	−5.2	mmol/L	Base deficit
BE(B)	0.6	mmol/L	Near normal
P/F Ratio	237	mmHg	Mild hypoxemia if on high FiO_2_
HCO_3_− (c)	13.9 ↓	mmol/L	Low (metabolic component)
HCO_3_− std	25.4	mmol/L	Near normal
Hct (c)	54 ↑	%	Elevated

^p^H: Hydrogen ion concentration; pCO_2_: Partial pressure of carbon dioxide; pO_2_: Partial pressure of oxygen; Na^+^: Sodium; K^+^: Potassium; Cl^−^: Chloride; Ca^2+^: Ionized calcium; O_2_Hb: Oxyhemoglobin; COHb: Carboxyhemoglobin; MetHb: Methemoglobin; HHb: Deoxyhemoglobin; SpO_2_: Oxygen saturation; TCO_2_: Total carbon dioxide; BEecf: Base excess in extracellular fluid; BE(B): Base excess in blood; P/F ratio: Ratio of PaO_2_ to FiO_2_; FiO_2_: Fraction of inspired oxygen; HCO_3_− (c): Calculated bicarbonate; HCO_3_− std: Standardized bicarbonate; Hct (c): Calculated hematocrit.
